# Extended approach to the lateral tibial plateau with central meniscal subluxation in fracture repair: feasibility and first clinical and radiographic results

**DOI:** 10.1007/s00068-020-01467-1

**Published:** 2020-08-31

**Authors:** Alexander Korthaus, Tobias Malte Ballhause, Jan-Philipp Kolb, Matthias Krause, Karl-Heinz Frosch, Maximilian J. Hartel

**Affiliations:** 1 Department of Trauma and Orthopaedic Surgery, University Medical Center Hamburg-Eppendorf, Martinistrasse 52, 20246 Hamburg, Germany; 2 Department of Trauma, Orthopaedic Surgery and Sports Traumatology, BG Trauma Hospital Hamburg, Hamburg, Germany

**Keywords:** Tibial plateau fracture, Meniscus subluxation, Extended approach, Articular surface, Open reduction internal fixation

## Abstract

**Purpose:**

Anatomic reduction in tibial plateau fractures remains to be demanding. For further visualisation of and approach to the joint surface an extended lateral approach using a lateral femoral epicondyle osteotomy and subluxation of the lateral meniscus was recently described. First clinical and radiographic mid-term results of this technique are presented in this feasibility study.

**Method:**

Ten complex tibial plateau fractures treated with extended lateral approach and lateral meniscal subluxation were prospectively analysed. Clinical and radiographic results were objectified according to the Rasmussen scores.

**Results:**

After a median follow-up of 8.6 (IQR 4.3) months good to excellent clinical and radiographic results were noted. The clinical Rasmussen Score showed a median of 25 (IQR 2.8) and radiographic a median of 17 (IQR 2.0) points.

**Conclusion:**

Good to excellent clinical and radiological scores were obtained after using an extended lateral approach with lateral femoral epicondyle osteotomy and central meniscus subluxation. No approach specific complications could be observed.

**Electronic supplementary material:**

The online version of this article (10.1007/s00068-020-01467-1) contains supplementary material, which is available to authorized users.

## Introduction

Tibial plateau fractures are amongst the uncommon fracture types with a proportion of less than 1% of all bony injuries [1]. Surgical therapy remains to be demanding as in most of the fractures with joint affection, the exact anatomic reduction is paramount [2]. Gaps and steps of more than 2 mm and varus or valgus deviations of more than 5° are significantly associated with impaired outcome [3]. However, articular malreductions after tibial plateau fracture fixation may still remain in more than 70–89% of the cases [4, 5]. Key factors to successful anatomic reduction are the thorough preoperative planning, resulting in the selection of the appropriate surgical approach. Failure to obtain accurate fracture visualization during surgery will increase the risk of malreductions [4, 6]. With the standard anterolateral approach, only 1/3 of the lateral tibiaplateau can be exposed [7]. For improved visualization extended approaches are described, with or without osteotomy of the fibula or osteotomy of the lateral femoral epicondyle [8–11].

Recently, a novel technique permitting improved visualization was introduced utilizing a lateral femoral epicondyle osteotomy [12, 13]. For further improvement of visualization, it is possible to dissect the lateral meniscus from the posterolateral capsule allowing for subluxation of the lateral meniscus into the intercondylar femoral notch [14]. However, clinical follow-up data were missing to date. In the present feasibility study, we aimed to describe the first clinical and radiological results of complex tibial plateau fractures treated by extended lateral approach including lateral femoral epicondyle osteotomy and lateral meniscal subluxation.

## Methods

The first ten patients treated with the new technique were included in this feasibility study and prospectively analyzed. There were four female and six male patients. The median age of the cohort was 59 years (IQR 4.7 range 27–90). The fractures were classified using the AO Classification [15] and the ten-segment classification [16]. The clinical and radiological Rasmussen-Scores were determined in all patients. The clinical Rasmussen-Score includes the following items: range of motion, collateral ligament stability, extension limitation, walking range, and pain. A score of 30 is interpreted as normal function, 27–30 as excellent, 20–26 as good, 10–19 as fair, and 6–10 as poor. The radiological Rasmussen-Score includes depression of the tibial plateau, condylar enlargement, and axial deviations. A score of 18 is interpreted as excellent, 12–17 as good, 6–11 as fair, and 0–5 as poor. Small-fragment locking plate systems were used in all of the cases [standard lateral plate: Smith&Nephrew PeriLoc (Hamburg, Germany), for posterior medial aspects Synthes LCP 3.5 mm T-plates, (Oberdorf, Switzerland) and posterolateral depending on size and fracture type a variety of differently sized T-locking plates produced by Synthes (3.5 mm or 2.4 mm) or Stryker (2.7 mm Variax Foot; Stryker Trauma GmbH, Schönkirchen, Germany).

### Surgical technique

**Fig. 1:**
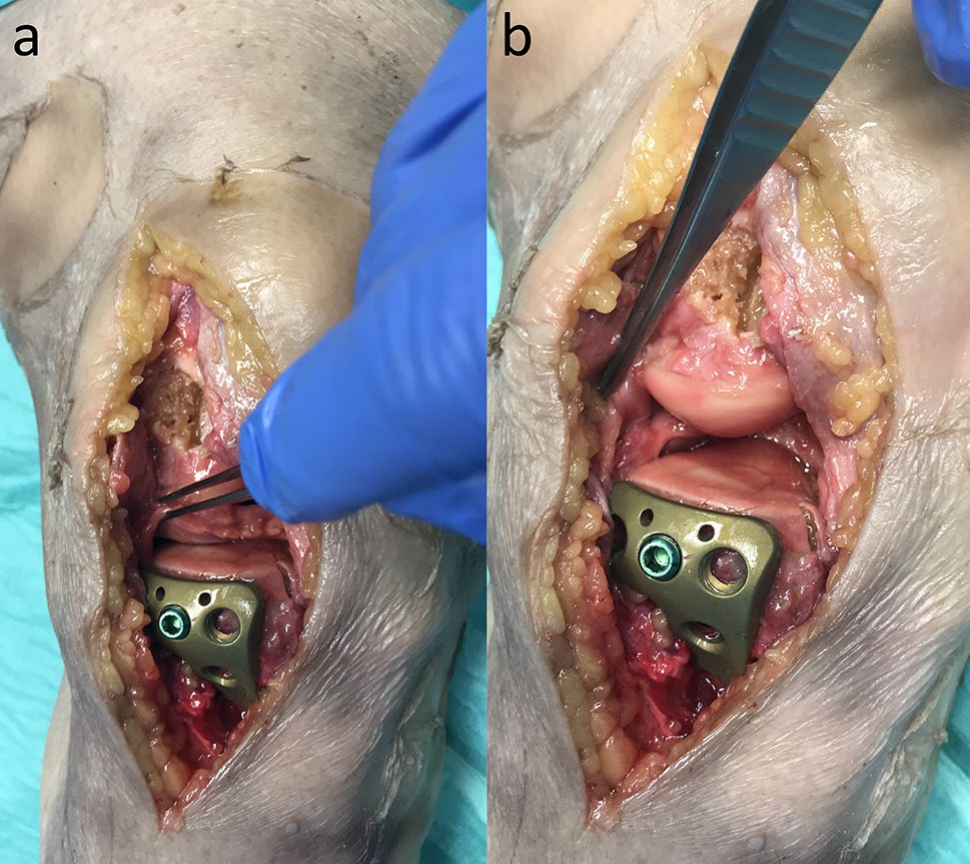
Demonstrates the effect of a lateral epicondyle osteotomy (**a**) and the additional effect of the central meniscal subluxation with a complete exposure of the lateral articular surface (**b**)

**Fig. 2:**
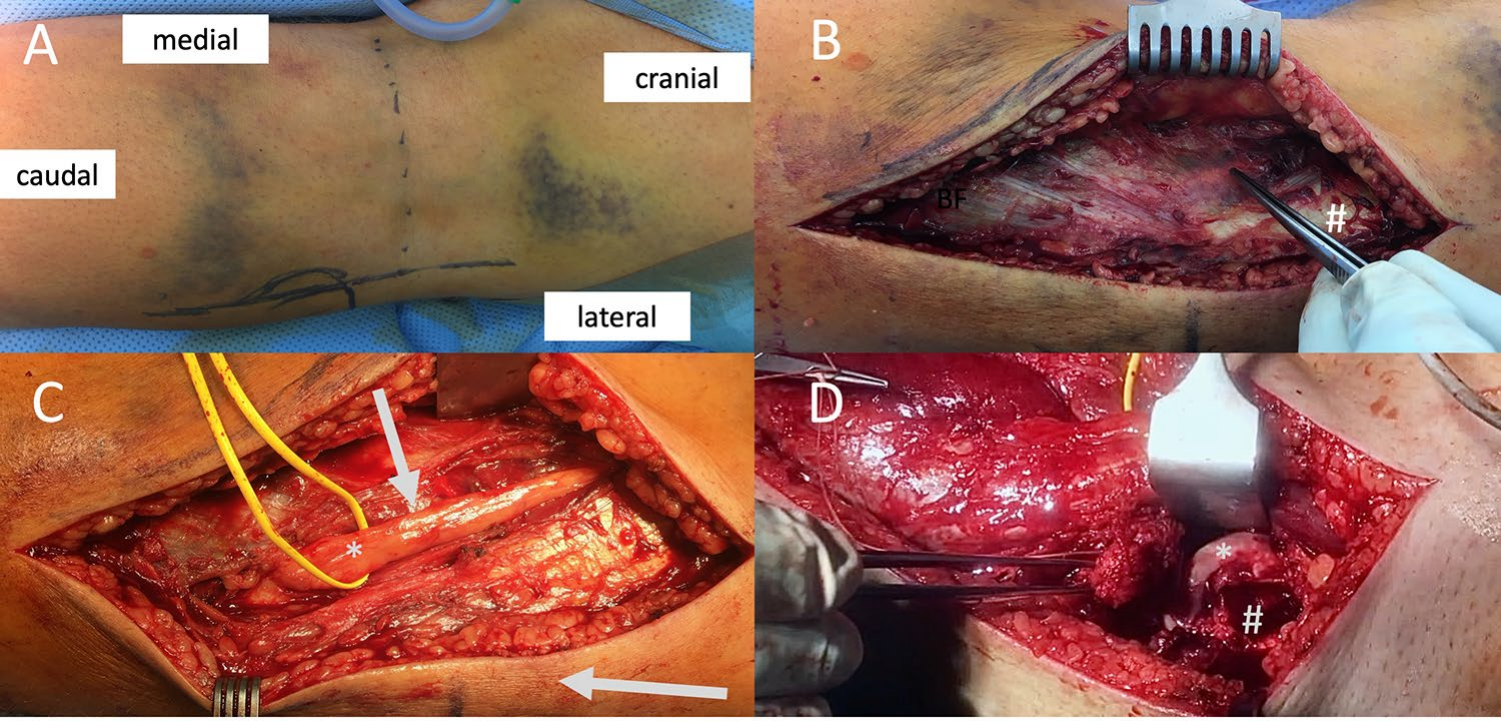
Demonstrate the operative procedure step-by-step in prone position: **a** Dashed line = popliteal skin crease, lateral vertical line = incision. **b** Epifascial preparation with identification of the peroneal nerve shining through from below the facsica, located usually just medial to the biceps femoris muscle (#). **c** Shows the peroneal nerve (*) dissected free and neurolysed. The arrows show the intervals to both, the posterior-lateral and anterior lateral approaches (picture taken before further epifascial dissection into anterior direction to Gerdy’s tubercle, where a second fascial incision is usually carried out). **d** The forceps holds the femoral epicondyle with the popliteal tendon and LCL attatched to it. *Lateral femoral joint surface, #Footprint of the femoal epicondyle osteotomy

**Fig. 3:**
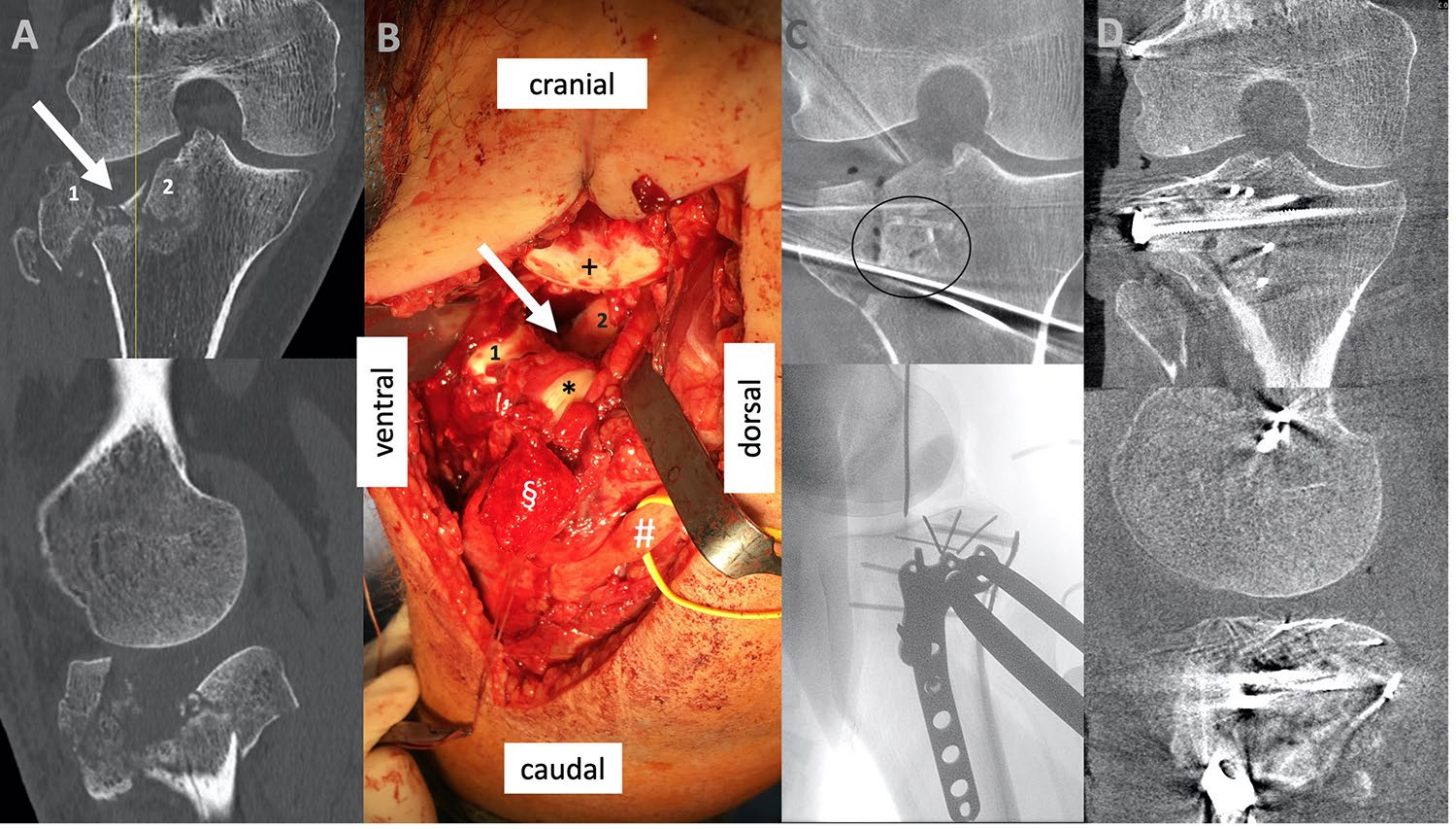
Shows preoperative and intraoperative images of the same case as shown in (Fig. 2), operated on in prone positioning, **a** Preoperative CT scan showing a comminuted lateral tibial plateau fracture. The arrow indicates the main depression, and two main fragments are numbered, with 1 the anterior-lateral main fragment and 2 a part of the centrally depressed main fragments. **b** Intraoperative situs (with # peroneal nerve, § osteotomized epicondyle fragment with * popliteus tendon and LCL attached. + Marks the lateral femoral condyles joint surface). The arrow and numbers 1 and 2 show once again the depression and the two main fragments corresponding to the markings on **a**; **c** Intraoperative coronar 3D scan after allograft implantation and first preliminary reduction attempt secured with wires, below a lateral fluoroscopic image which shows the double plate placement; **d** Final intraoperative 3D scan after allograft implantation and finalized ORIF

The extended lateral approach with central meniscal subluxation was indicated according to the concept of direct approach to the fracture and stepwise extension as needed. Due to this concept an extension of the lateral approach was performed by an osteotomy of the lateral femoral epicondyle if a complete exposure of the fractured articular segments of the tibial plateau could not be achieved during surgery. And only if the osteotomy of the lateral femoral epicondyle was not enough to achieve a full visualization of the fracture, an additional central subluxation of the lateral meniscus was performed. In all cases in this study the extended lateral approach as described by Frosch et al. [9, 10] combined with an epicondyle osteotomy and a central meniscus subluxation was performed (Fig. 1). A longitudinal incision was carried out lateral in supine or posterolateral in prone position, starting 3 cm proximal the joint line. The incisions length is usually around 12–15 cm (Fig. 2). Deep subcutaneous dissection was followed by a longitudinal incision of the iliotibial band directly over the lateral femoral epicondyle with the knee flexed at 30°–40°. The peroneal nerve was visualized, dissected and carefully protected. The ilio-tibial band and the fascia of the m. tibialis anterior were released from the anterior edge of the fibula head from Gerdy’s tubercle. The lateral collateral ligament (LCL) was displayed to the bony insert at the lateral femoral epicondyle. Osteotomy of a bone block (2 × 2 × 1 cm) of the lateral femoral epicondyle with including the femoral origin of the popliteus tendon and the LCL was performed. After incision of posterolateral meniscocapsular fibers, around 5 mm away from the meniscal rim the lateral meniscus was centrally subluxated (Figs. 1, 3). At the end of the case the meniscus was reattached to its anatomical position using Vicryl sutures (size 0). The meniscal roots and the anterior capsule of the lateral meniscus stayed intact.

In 4 cases an additional posteromedial approach was required for ORIF of the medial tibial condyle in bicondylar fractures. All bicondylar fractures were treated in prone position with an additional longitudinal skin incision above the medial head of the gastrocnemius muscle. Subsequently, the fascia above the muscle is incised and the medial head of the gastrocnemius was bluntly mobilized and retracted to the lateral side. Depending on the fracture location and required hardware positioning a thrifty dissection of the soleus and popliteus muscle attachments was carried out.

## Results

All the fractures treated with the novel extended approach including central meniscal subluxation had an involvement of the postero-lateral segments. In all but one of the cases a fragmentation in the posterolatero-central segment was encountered. There were four bicondylar fractures requiring an additional posteromedial approach (Table 1). There was no polytraumatized patient in the cohort.

**Table 1:** Classification and follow up of the cohort

Sex	Age^a^	Type of fracture (AO-classification)	Type of fracture (Ten-segment-classification)	Approach	FU Time^b^	rR	cR
♂	54	41B3.3	ALL;ALC;PLL;PLC;PC	XL, PM	12.2	16	24
**♀**	27	41C3.3	ALL;ACL;PLL;PLC;PC;AC;PMC;PMM	XL, PM	5.3	16	26
**♂**	56	41B3.3	PLL,PLC,PC	XL	6.7	18	28
**♀**	62	41B3.3	ALL;ALC;PLL;PLC	XL	4.4	18	26
**♀**	59	41C3.3	ALL;ALC;PLL;PLC;PC	XL	2.8	17	25
**♀**	67	41C3.3	ALL;ACL;PLL;PLC;PC;AC;PMC;PMM	XL, PM	7.9	17	23
**♀**	90	41B3.3	ALL,PLL,PC	XL	9.3	18	29
**♂**	58	41B3.3	ALL,PLL; ALC,PLC,PC	XL	9.5	16	25
**♀**	58	41C3.3	ALL;ACL;PLL;PLC;PC;PMC;PMM	XL	10.2	18	21
**♂**	59	41C3.3	ALL;ACL;PLL;PLC;PC;AC;PMC;PMM	XL, PM	14.5	15	22

*AMM* antero-medio-medial, *AMC* antero-medio-central, *PMM *postero-medial-medial, *PMC* postero-medial-central, *AC* antero-central, *PC* postero-central, *ALL* antero-latero-lateral, *ALC* antero-latero-central, *PLL* postero-latero-lateral, *PLC* postero-latero-central, *rR* radiological Rasmussen score, *cR* clinical Rasmussen score, *XL* described extended lateral approach, *PM* posteromedial approach

^a^In years

^b^In months

In six out of the ten cases the intraoperative 3D-scans (Siemens Cios Spin) were performed to ensure anatomic reduction and adequate positioning of fixation devices. In two of these 6 cases the 3D scan led to an intraoperative revision.

In median 5 (IQR 5.3 range 3–10) segments according to the ten-segment classification [16] were involved.

In the outpatient follow-up visits, excellent and good clinical and radiologic results were encountered in all of the cases after a median follow-up of 8.3 (IQR 4.3 range 3–14.5) months. None of the ten cases required a post-operative revision surgery.

The clinical Rasmussen-Score had a median value of 25 (IQR 2.8, range 21–29). Two patients (20%) had excellent (with score > 27) and the eight (80%) had good (score 20–26) clinical results.

Just one patient had a limited walking range with less than 15 min and a ROM of less than 90° at a follow-up of 10.2 months. He had an excellent radiological Score of 18. The analysis of the follow-up radiographs revealed a median score of 17 (IQR 2.0 range 15–18). Two cases had a joint gap of 2 and 2.3 mm without articular step. Forty percent of the cases had an excellent (18 points) and 60% had good (12–17 points) radiological score (Table 3).

There were no significant differences between the bicondylar and unicondylar tibial plateau fractures (Tables 2, 3).

**Table 2:** Clinical rasmussen score

	All cases	Bicondylar	Unicondylar
	Median (IQR)	Median (IQR)	Median (IQR)
Subjective			
Pain	5.0 (1.5)	4.5 (1.3)	5.0 (0.8)
Walking capacity	4.0 (1.5)	4.0 (1.0)	4.0 (1.5)
Objective			
Extension	4.0 (2.0)	4.0 (0.5)	5.0 (2.0)
Total range of motion	5.0 (0.8)	4.5 (1.0)	5.0 (0.8)
Stability	6.0 (0.0)	6.0 (0.3)	6.0 (0.0)
Total	25.0 (2.8)	22.5 (2.0)	25.0 (2.5)

**Table 3:** Radiological rasmussen score

	All cases	Bicondylar	Unicondylar
	Median (IQR)	Median (IQR)	Median (IQR)
Depth	6.0 (0.0)	6.0 (0.5)	6.0 (0.0)
Width	6.0 (0.8)	5.5 (1.0)	6.0 (0.0)
Angulation	6.0 (0.0)	6.0 (0.5)	6.0 (0.0)
Total	17 (2.0)	16.5 (1.5)	17.0 (1.8)

Meniscal signs during follow up were negative in all patients. No approach specific complications could be observed.

## Discussion

The first clinical and radiologic results utilizing the novel technique of lateral femoral epicondyle osteotomy combined with lateral meniscal subluxation are promising with good to excellent results even in the presented subselection cases with of most complex lateral tibial plateau fractures. Compared to previously published work the results presented in the study are at least equal or superior. Former case series showed not only excellent and good clinical and radiological results according to the Rasmussen score but also fair and poor results, too [17, 18]. Jiang et al. treaded Schatzker II and Schatzker V/VI fractures with an extended anterolateral approach and reached similar results. However posterolateral fragments could not be addressed in 16.7% [19]. In postoperative analyses of Schatzker II and III treated by an extended approach with a fibular head osteotomy they had excellent functional and radiological Rasmussen-Score results, but one peroneal nerve injury in a mean follow-up of 3.2 years [20].

The cohort analyzed included complex fracture patterns with involvement of the central as well as postero-lateral-central segment of the lateral tibial plateau. This is consistent with the research of Krause et al. which showed that in 84.7% of type C injuries the postero-lateral-central segment is involved [16]. These areas are particularly difficult to visualize and reduce [21]. Meulenkamp et al. described a revision rate up to 77% for fractures of the lateral tibial plateau [4, 21]. In the cases presented, the institution’s concept of using a stepwise extension of the surgical approach as needed was applied. The fractures were approached directly using well established standard approaches. However just 36% of the lateral tibial plateau can be viewed through the anterior lateral standard approach [11]. If required, in a second step an epicondyle osteotomy was carried out for improved visualization of the lateral plateau. As previous work showed, this extension permits the visualization of up to 83% of the lateral plateau [22]. Up to almost 100% of the lateral plateau may be visualized by that way [14]. The meniscal subluxation and the approach extension should be performed with care and only if a complete visualisation of the fracture is required in cases of massive comminution. This stepwise expansion helps to stay as less invasive as individually possible. In the first ten patients with complex tibial plateau fractures treated with the central meniscal subluxation the postero-lateral section needed to be addressed in every case. To complete the reduction and fixation needed in these particular cases, all surgical steps, including lateral epicondyle osteotomy and meniscal subluxation were required. In the case series presented, there was no necessity for revision surgery.

Several other approaches are described that allow to address the posterior aspect of the lateral plateau: It is possible to osteotomize the lateral tibial condyle, especially useful in such cases, where an osteotomy completes a split component. This technique allows direct desimpaction and grafting of the depressed joint line [23]. Moreover, as Hu described, it is possible to get access further posteriorly using a modified anterior-lateral supra-fibular approach, in which the LCL is identified and retracted with the knee in a 60° flexion [24]. Next to a potential risk for iatrogenic LCL injury, there are two drawbacks of such concepts: first, approaches, that include an Osteotomy for the release of lateral ligamentous structures allow direct inspection of the joint not only prior to, but also after completion of the reduction of a depressed joint line. This fact likely leads to superior reduction results. For example, after flipping back the osteotomized tibial condyle, it is challenging to exactly double check on the final reduction result centrally. An intraoperative 3D scan or fracturoscopy is required in such a situation to be sure [5, 25–27]. Secondly, the extended lateral approach as decribed in this paper, especially if carried out in prone positioning, allows direct access to the posterior-lateral corner from posteriorly. This way, not only a direct manipulation of a posterior-lateral-central fragment from posterior, but also direct grafting and butressing using a posterior-lateral plate is made possible (e.g., Fig 3).

When compared with the extended approach with fibular osteotomy, the femoral epicondyle osteotomy has several advantages such as lower invasivity and likely lower approach related morbidity and lower complication rates [9, 22, 28, 29].

Finally, one major approach related technical advantage exists with the femoral epicondyle osteotomy: as not only the LCL, but also the popliteus tendon is released, the relaxation of the popliteal tendon allows further retraction of the popliteus muscle posteriorly, which leads to a significantly improved exposure at the posterior-lateral window. This additional space is gladly taken for further facilitated reduction, grafting and fixation.

In six of the cases 3D imaging was utilized. In two of these, intraoperative revisions were indicated with additional reduction maneuvers carried out during the same procedure. In a larger series of 559 cases Beisemann et al. demonstrated similar numbers with a 26.5% intraoperative revision rate [26].

There are several limitations to this study: the sample size was small including a wide age distribution and different, but complex fracture patterns. There was no comparison group. This work’s primary intention however, was to report on the first midterm results in the first consecutive case series in which this novel extensile approach was used. The early results of the first 10 patients show consistently high score values indicate promising long-term results. However, long-term outcomes and complication rates will have to be determined in a larger cohort in the future.

The authors would like to point out, that an extended approach as described in this study should only be used, where indicated to minimize iatrogenic soft tissue damage.

In conclusion, excellent to good postoperative mid-term results can be expected after the use of an extended lateral approach with epicondyle osteotomy and meniscus subluxation for improved visualization of the lateral tibial plateau. No complications and no need for revision were encountered in the first consecutive patient series of ten. Larger studies with long-term follow-up are required for final clarification.

## Electronic supplementary material

Below is the link to the electronic supplementary material.

Supplementary file1 (MP4 72625 kb)
